# The competent sentinel node: an association with an axillary presentation and an occult or a small primary invasive breast carcinoma

**DOI:** 10.1186/1477-7800-3-39

**Published:** 2006-11-21

**Authors:** Lucy Mansfield, Haresh Devalia, Nadeem Rehman, Kefah Mokbel

**Affiliations:** 1 St. George's Hospital, London, UK; 2 The Princess Grace, Hospital, London, UK

## Abstract

The concept of the sentinel node describes a primary or sentinel lymph node (SLN), which exists and through which tumour cells from a primary tumour in a particular location must first travel to spread to a particular regional lymph node group. In this series we present three patients presenting with a pathological axillary node associated with either an occult or very small primary breast cancer. In each case the primary tumour was found to have metastasised to the palpable node, however despite the significant enlargement of this node, no other axillary nodes were found to be affected on axillary node clearance. This has led us to postulate that the SLN in some cases contains unique characteristics that enable it to prevent further spread of the tumour up the lymphatic chain. Hence the term the competent sentinel node.

## Background

The concept of the sentinel node describes a primary or sentinel lymph node (SLN), which exists and through which tumour cells from a primary tumour in a particular location must first travel to spread to a particular regional lymph node group.

Recent studies have demonstrated that the sentinel node biopsy (SNB), which utilizes a simple principle, is a reliable and minimally invasive method for determining the status of the regional lymph nodes in patients with clinically node-negative breast cancer [1].

In this series we present three patients presenting with a pathological axillary node associated with either an occult or very small primary breast cancer. In each case the primary tumour was found to have metastasised to the palpable node, however despite the significant enlargement of this node, no other axillary nodes were found to be affected on axillary node clearance. This has led us to postulate that the SLN in some cases contains unique characteristics that enable it to prevent further spread of the tumour up the lymphatic chain. Hence the term the "competent sentinel node".

## Case 1 (NL)

**Figure 1:**
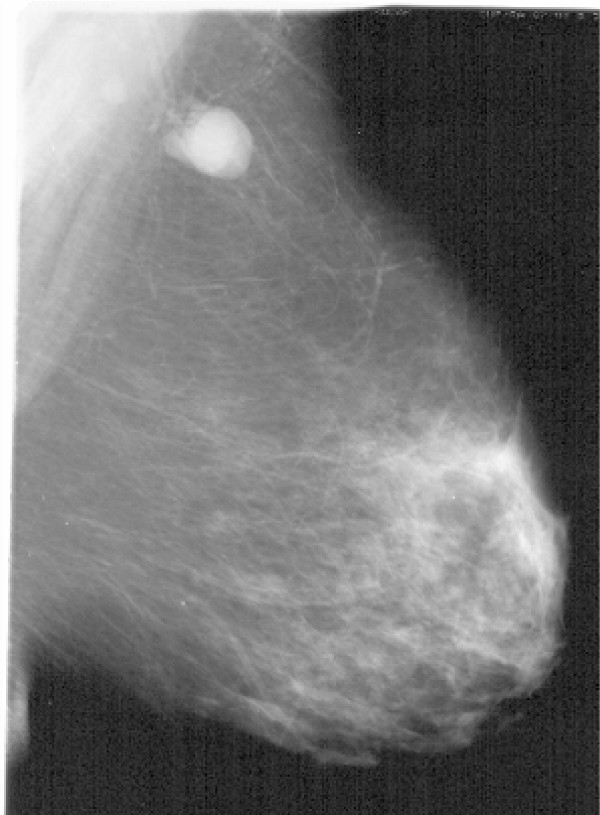
Title: Mammogram from case 1. Data description: Mammogram confirms the presence of a pathological lymph node with no evidence of a breast primary lesion.

This 53-year-old lady presented with a pathological lymph node in the left axilla measuring approximately 3 cm. No other abnormalities were noted. Mammography, ultrasound examination and MRI scanning confirmed the presence of a pathological lymph node with no evidence of a breast primary lesion (fig. 1). In addition a CT scan of the thorax again showed the solitary axillary lymph node however no other abnormalities. A fine needle aspiration cytology (FNAC) of this node was reported as metastatic breast carcinoma. (ER, PgR, CerbB2, positive). It was decided at this point to proceed to a level I-III axillary lymph node dissection. Pathological examination revealed only 1/14 axillary nodes showing metastatic carcinoma consistent with a breast primary. This node measured 38 mm in greatest dimension (ER 8/8, PgR 8/8, CerbB2 2+). There was no extracapsular spread and all adjacent fatty tissue showed normal duct-lobular breast units. The patient was subsequently treated with chemotherapy, breast irradiation and endocrine therapy.

## Case 2 (SM)

This 33-year-old lady presented with a pathological lymph node in the right axilla measuring approximately 3 cm. No other abnormalities were noted. Mammography, ultrasound examination and MRI scanning confirmed the presence of a pathological lymph node (30 mm MRI size). The USS showed an indeterminate area in the lateral aspect of the right breast which was confirmed to be the primary lesion on core biopsy. An FNAC of the lymph node was reported as metastatic breast carcinoma. It was decided at this point to proceed to a level I-III axillary lymph node dissection combined with skin-sparing mastectomy and immediate reconstruction. Pathological examination revealed a 4 mm a primary breast carcinoma (grade 3 infiltrating ductal, negative for ER, PgR and Her-2) and only 1/21 axillary nodes showed metastatic carcinoma. There was only very scanty extracapsular spread. The patient was subsequently treated with chemotherapy.

## Case 3 (JW)

This 48-year-old lady presented with a pathological lymph node in the right axilla measuring approximately 3 cm. No other abnormalities were noted. Mammography, ultrasound examination and MRI scanning confirmed the presence of a pathological lymph node (35 mm MRI size). No primary lesion was identifies. An FNAC of the lymph node was reported as metastatic breast carcinoma. It was decided at this point to proceed to a level I-III axillary lymph node dissection. Pathological examination revealed only 1/10 axillary nodes showing metastatic carcinoma consistent with a breast primary. This node measured 35 × 25 × 20 mm. E-cadherin was positive consistent with a ductal carcinoma of the breast. The HER-2 FISH result revealed a ratio of 0.89. Her treatment was completed with chemotherapy and radiotherapy to the breast. Two years later she continues to take tamoxifen and there has been no evidence of recurrence.

## Discussion

The history of breast cancer management during the last few decades has been one of decreasing invasiveness in order to try and achieve a much lower level of morbidity. To help reach this improved level of treatment the concept of the SLN was utilized.

This concept describes a primary or sentinel lymph node (or nodes), which exists and through which tumour cells from a primary tumour in a particular location must first travel to spread to a particular regional lymph node group. These sentinel nodes are therefore the nodes most likely to harbor tumour cells if the cancer has indeed entered the lymphatics. A tracer substance (a radioactive isotope and blue dye) injected into the breast provides a roadmap leading to the SLN(s).

The SLN concept was initially introduced by Cabanas in 1977 [2]. He applied it to the management of penile cancer. The technique was popularized in the 1980s and early 1990s in the management of melanoma by Morton et al [3]. They used the injection of isosulfan blue dye to allow visualisation as it flowed through, and stained lymphatic channels and nodes, enabling identification, excision and assessment of blue-stained nodes. In the early 1990s the SLN concept was applied to the management of breast cancer patients. These initial efforts followed the progress made in the management of melanoma, predominantly with the use of both radiotracer and blue-dye techniques to help identify the sentinel node. The SNB has now become the gold standard of assessing the invasion of tumour into the lymphatics in patients with clinically node-negative invasive breast cancer with a much lower operative morbidity than the previously routine axillary nodal dissections [1,4].

It is well recognised that intravasation of tumour cells into the vasculature and/or lymphatics is a key stage in the metastatic process, and the technique of sentinel node biopsy has provided confirmation of the orderly anatomic progression of tumour cells from the primary site to the regional lymph nodes through lymphatic capillaries and trunks [5]. There remains very little understanding in regards to the mechanisms involved in these events. Less is known about the methods of lymphatic intravasation in comparison to vascular invasion. However the process of lymphangiogenesis is thought to be similar to the well-known mechanism of angiogenesis. Molecular research techniques have suggested that likely factors involved include tumour-secreted cytokines such as vascular endothelial growth factors, tumour cell expression of specific chemokine receptors, adhesion molecules and integrins [5,6]. The directional movement towards lymphatics and lymph nodes appears to follow a chemokine environment [5].

From our case series we have developed the theory of the "competent sentinel node". Interestingly, all the cases had axillary presentation and were associated with either an occult or very small primary tumour. Examination of the nodes invaded with tumour cells, revealed them all to be of a significant size without any significant extra-capsular spread. More importantly in each axillary nodal clearance no other nodes were found to be affected, despite the significant size and tumour load within the affected node which is by definition is the true SLN. A study by Wada et al supports our findings, suggesting that a small primary tumour size is less likely to be associated with involvement of non-SLNs [7].

This raises the question in regards to the competency of the sentinel node. Our concept of competent SLN is also consistent with the observation that the SLN is the only node involved in approximately two thirds of node positive patients [1,4]. There appears to be some inherent ability within the SLN in such cases that prevents spread of the tumour further up the lymphatic chain and this could be indicative of good prognosis. The sentinel node appears to provide a favorable molecular micro-environment which allows the tumour cells to survive and proliferate. Further studies and research needs to be performed in order to help examine what determines this competence, if indeed it does exist. The anatomical efferent lymphatic pathway, tumour pathology and local biological factors within the lymph nodes involved may help provide an answer to help explain the ability of the sentinel node to contain further tumour spread, i.e. its competency. This is obviously an area that requires much more research in order to create a much greater understanding in regards to the properties of the sentinel node and the exact mechanisms behind tumour spread.

## Competing interests

The author(s) declare that they have no competing interests.

## Authors' contributions

LM carried out overall design of the manuscript and was responsible for all literature reviews and the writing of the article itself.

HD contributed towards the literature review and discussion

NR contributed towards the case presentations

KM conceived the theory behind the article and helped to draft the manuscript
